# Intravesical Delivery of P21 mRNA–Loaded Lipid Nanoparticles as a Tumor Suppressor Replacement Therapy for Bladder Cancer

**DOI:** 10.1096/fj.202600049R

**Published:** 2026-05-17

**Authors:** Jie Zeng, Ziyi Cao, Chenghe Wang, Yingjie Xu

**Affiliations:** ^1^ Department of Biochemistry and Molecular Cell Biology, Shanghai Key Laboratory for Tumor Microenvironment and Inflammation Shanghai Jiao Tong University School of Medicine Shanghai People's Republic of China; ^2^ Key Laboratory of Cell Differentiation and Apoptosis of the Chinese Ministry of Education Shanghai Jiao Tong University School of Medicine Shanghai People's Republic of China; ^3^ Department of Urology, Ruijin Hospital Shanghai Jiao Tong University School of Medicine Shanghai People's Republic of China; ^4^ Shanghai RNACure Biopharma Co. Ltd Shanghai People's Republic of China

**Keywords:** bladder cancer, CDKN1A/p21, Intravesical delivery, lipid nanoparticles, mRNA therapy, tumor suppressor replacement

## Abstract

Bladder cancer is characterized by high recurrence rates and limited long‐term benefit from current intravesical therapies, highlighting the need for alternative localized treatment strategies. Among tumor suppressors altered in bladder cancer, *CDKN1A*, which encodes the cyclin‐dependent kinase inhibitor p21, is recurrently inactivated and downregulated, supporting its potential as a target for tumor suppressor replacement. Here, we developed a non‐viral therapeutic strategy based on chemically modified p21 mRNA encapsulated in lipid nanoparticles (p21‐LNP) for intravesical delivery. Public dataset analysis, tissue microarray staining, and cell line validation showed that p21 expression decreases during bladder cancer progression and that endogenous p21 protein levels are very low in bladder cancer cells. In vitro, synthetic p21 mRNA achieved robust nuclear p21 expression and markedly suppressed bladder cancer cell proliferation, viability, and clonogenicity. Mechanistically, p21 restoration reduced retinoblastoma protein (Rb) phosphorylation, decreased Cyclin E, Cyclin B, and proliferating cell nuclear antigen (PCNA) expression, increased γ‐H2A.X accumulation, and promoted apoptosis. The resulting p21‐LNP showed favorable physicochemical properties for intravesical administration. In vivo, reporter mRNA‐LNP mediated strong bladder‐localized protein expression with limited and transient systemic distribution. In an orthotopic bladder cancer mouse model, repeated intravesical administration of p21‐LNP significantly suppressed tumor growth, restored p21 expression in bladder tissues, and preserved urothelial architecture without obvious adverse effects. Together, these findings establish intravesical delivery of p21 mRNA‐LNP as a clinically compatible strategy for localized tumor suppressor replacement therapy in bladder cancer.

## Graphical abstract

Intravesical delivery of p21 mRNA‐loaded lipid nanoparticles (p21‐LNP) restores the tumor suppressor p21 in bladder cancer. Localized p21 expression directly induces cell cycle arrest, DNA damage and apoptosis, and ultimately inhibits orthotopic tumor growth with minimal systemic distribution and favorable safety. This strategy provides a clinically compatible approach for tumor suppressor replacement therapy in bladder cancer.

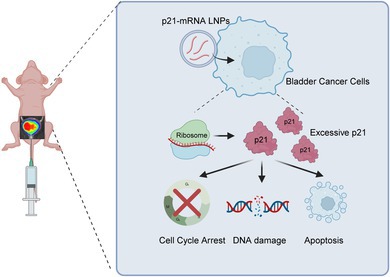

## 1 Introduction

Bladder cancer is a common malignancy of the urinary tract with high rates of recurrence and progression [1, 2]. Approximately 70%–75% of newly diagnosed cases are non–muscle‐invasive bladder cancer (NMIBC), for which intravesical therapy is the standard treatment and enables localized drug delivery with limited systemic toxicity [2]. However, current intravesical approaches, including chemotherapy and Bacillus Calmette–Guérin (BCG) immunotherapy, are frequently limited by resistance, incomplete response, and adverse effects [3, 4]. New localized therapeutic strategies are therefore needed.

Messenger RNA (mRNA) has emerged as a promising therapeutic platform for vaccines, protein replacement, and cancer treatment [5, 6, 7]. In vitro transcribed (IVT) mRNA does not integrate into the host genome, is naturally degraded in vivo, and can be rapidly manufactured, making it attractive for transient therapeutic protein expression [5]. However, the broader application of mRNA therapeutics in non‐hepatic solid tumors remains constrained by delivery. Following systemic administration, lipid nanoparticles (LNP) preferentially accumulate in the liver, which limits drug exposure at extrahepatic tumor sites and narrows the therapeutic window [8, 9, 10]. For tumors amenable to direct local administration, localized mRNA delivery may therefore offer a more practical translational strategy.

The bladder is especially well‐suited for localized mRNA delivery. As a hollow organ accessible through a clinically established catheter‐based instillation route, it permits direct exposure of therapeutic formulations to urothelial tumors while minimizing systemic dissemination [11, 12]. This route is also well matched to the transient expression profile of mRNA, because repeated intravesical dosing is already routine in bladder cancer management [2, 11].

Among candidate tumor suppressors altered in bladder cancer, *CDKN1A*, which encodes the cyclin‐dependent kinase inhibitor p21, is supported by strong genomic and clinical evidence as a biologically relevant target. Prior studies identified recurrent inactivating *CDKN1A* mutations in bladder cancer and linked disruption of the *TP53*/*CDKN1A*/*CDKN2A*/*MDM2* axis to invasive disease [13]. Comprehensive molecular profiling of muscle‐invasive bladder cancer further showed that the p53/cell‐cycle pathway is altered in the vast majority of cases, with *CDKN1A* mutations present in a substantial subset of tumors and predominantly inactivating [14]. These observations position p21 as a clinically relevant tumor suppressor in bladder cancer. p21 is mechanistically well‐suited for mRNA replacement. As a direct inhibitor of cyclin–cyclin‐dependent kinase (CDK) activity, restored p21 can rapidly suppress Rb phosphorylation, restrain cell‐cycle progression, and trigger growth arrest and apoptosis in highly proliferative tumor cells [15, 16]. Unlike upstream pathway restoration strategies, p21 acts directly on downstream cell‐cycle machinery and may therefore remain effective in tumors with defective p53 signaling [16, 17, 18].

Here, we developed an intravesical tumor suppressor replacement strategy based on lipid nanoparticle–mediated delivery of p21 mRNA (p21‐LNP) for bladder cancer. We established the clinical relevance of p21 loss and demonstrated that exogenous p21 expression restores cell cycle control and suppresses tumor growth. Importantly, intravesical administration enabled localized delivery with minimal systemic exposure and favorable tolerability.

## 2 Materials and Methods

### 2.1 Plasmid Construction

The coding sequence of human p21 (*NM_000389*) was cloned into the pcDNA3.1 vector (General Biology). The EGFP coding sequence was synthesized and cloned into pSG5L (General Biology). A C‐terminal HA tag was added using the ClonExpress II One Step Cloning Kit (Vazyme, C112‐01). The sequences of all constructs were confirmed by Sanger sequencing.

### 2.2 In Vitro Transcription ( **IVT** ) of **mRNA**

Linearized DNA templates were generated by PCR using a forward primer containing the T7 promoter sequence and a reverse primer containing a 100‐nucleotide Poly (T) tract to encode the Poly (A) tail. The purified PCR products served as templates for IVT. Transcription was performed using the T7 RNA Polymerase Mix (APExBIO, K1083) supplemented with ATP (APExBIO, K1043), CTP (APExBIO, K1045), GTP (APExBIO, K1044), and N1‐methylpseudo‐UTP (APExBIO, B8049). EzCap (APExBIO, B8177) was included to facilitate co‐transcriptional 5′‐capping. Following incubation at 37°C for 3 h, the DNA templates were degraded by DNase I digestion (Thermo Fisher Scientific, EN0521) at 37°C for 15 min. The synthesized mRNA was purified using the RNA Clean & Concentrator kit (APExBIO, K1069), and its concentration was determined prior to downstream applications.

### 2.3 Cell Lines and Culture

The human bladder carcinoma cell line T24 was obtained from the Cell Resource Center, Shanghai Institutes for Biological Sciences, Chinese Academy of Sciences. The human bladder carcinoma cell line J82 and the human embryonic kidney cell line HEK293T were purchased from the American Type Culture Collection (ATCC). J82 and T24 cells were cultured in McCoy's 5A Medium (BasalMedia, L630KJ) supplemented with 10% fetal bovine serum (FBS) (Gibco, 10 099 141). HEK293T cells were cultured in Dulbecco's Modified Eagle's Medium (DMEM) (BasalMedia, L110KJ) supplemented with 10% FBS. All cells were maintained at 37°C in a humidified incubator containing 5% CO_2_. Cell lines were kept under sterile conditions and routinely tested for mycoplasma contamination.

### 2.4 Generation of Stable Cell Lines

HEK293T cells were co‐transfected with the pLenti‐Luc plasmid and packaging plasmids using polyethyleneimine (Polysciences, USA). Viral supernatants were harvested 48 h post‐transfection and used to infect newly plated T24 cells in the presence of polybrene to enhance transduction efficiency. Forty‐eight hours post‐infection, cells were subjected to selection with 2 μg/mL puromycin (APExBIO, B7587). Stable cell lines were established after 3–5 days of selection.

### 2.5 mRNA Transfection

Cells were seeded in 6‐well plates and transfected upon reaching 60%–70% confluence. For each well, 2 μg of mRNA and 4 μL of Lipofectamine 2000 (Thermo Fisher Scientific, 11 668 019) were diluted separately in 100 μL of Opti‐MEM medium (Thermo Fisher Scientific, 11 058 021) and incubated for 5 min at room temperature. The mRNA and the Lipofectamine mixture were combined, incubated for an additional 10 min, and added dropwise to the culture medium. All in vitro experiments were independently repeated at least three times.

### 2.6 Western Blotting

Cells or tissues were lysed in radio immunoprecipitation assay (RIPA) buffer (NCM Biotech, WB3100) supplemented with protease inhibitor cocktail (Roche, 04693159001) and 1 mM phenylmethylsulfonyl fluoride (PMSF) (Amresco, 0754). Lysates were clarified by centrifugation at 13 000 rpm for 20 min at 4°C. Protein concentrations were determined using a bicinchoninic acid (BCA) assay Kit (Sangon Biotech, C503021). Samples were mixed with SDS loading buffer (Epizyme, LT101S), denatured at 95°C for 10 min, separated by SDS‐PAGE, and transferred onto nitrocellulose membranes (Millipore, HATF00010).

Membranes were blocked with 5% non‐fat milk in Tris‐buffered saline containing 0.1% Tween 20 (TBST) and incubated overnight at 4°C with primary antibodies. Primary antibodies included: Anti‐p21 (CST, 2947, 1:1000), anti‐p21 (Abcam, ab188224, 1:1000), anti‐phospho‐Rb (Ser807/811) (CST, 8516, 1:1000), anti‐Cyclin E (CST, 20808, 1:1000), anti‐Cyclin B (CST, 12231, 1:1000), anti‐PARP1 (CST, 9542 T, 1:1000), anti‐γ‐H2A.X (CST, 9718S, 1:1000), anti‐PCNA (HuaBio, ET1605‐38, 1:2000), anti‐Vimentin (Sigma, V6389, 1:1000), anti‐VE‐cadherin (Wanleibio, WL02033, 1:1000), anti‐GAPDH (Proteintech, 81 640–5‐RR, 1:5000), anti‐β‐actin (Proteintech, 81 115–1‐RR, 1:5000), and anti‐EGFP (Proteintech, 66 002–1‐Ig, 1:10000).

After washing, membranes were incubated with horseradish peroxidase (HRP)‐conjugated secondary antibodies for 1 h at room temperature. Secondary antibodies included HRP‐Goat Anti‐Mouse IgG (H + L) (APExBIO, K1221, 1:10000), HRP‐Rabbit Anti‐Goat IgG (H + L) (APExBIO, K1224, 1:10000), and HRP‐Goat Anti‐Rabbit IgG (H + L) (APExBIO, K1223, 1:10000). Protein bands were visualized using an enhanced chemiluminescence (ECL) kit (Tanon, 180–501) and imaged with the ChampChemi system.

### 2.7 Immunofluorescence ( **IF** )

Cells were cultured on sterile glass coverslips in 6‐well plates. After transfection, cells were washed, fixed with 4% paraformaldehyde for 15 min, and permeabilized with 0.2% Triton X‐100. Non‐specific binding was blocked with a solution containing 2% goat serum and 2% bovine serum albumin (BSA) for 1 h. Cells were then incubated with anti‐HA primary antibody (CST, 3724S, 1:300) for 1 h, followed by fluorescent secondary antibodies for 30 min. Secondary antibodies used were goat anti‐mouse IgG (H + L) Alexa Fluor 488 (Invitrogen, A32723, 1:400) and goat anti‐rabbit IgG (H + L) Alexa Fluor 568 (Invitrogen, A32731, 1:400). Nuclei were counterstained with DAPI. Coverslips were mounted using ProLong Diamond Antifade Mountant (Invitrogen, P36961), and images were acquired using a Nikon ECLIPSE E800 fluorescence microscope. Image analysis was performed using ImageJ software.

### 2.8 Cell Proliferation and Viability Assays

Cells were harvested and counted using an automated cell counter (JIMBIO iCytal). For cell viability assays, cells were trypsinized 6 h post‐mRNA transfection and seeded into 96‐well plates at a density of 1 × 10^4^ cells/well. Cell viability was assessed at 24 h post‐transfection using the CCK‐8 Kit (APExBIO, K1018) according to the manufacturer's instructions. Absorbance at 450 nm was measured using a microplate reader (Tecan Infinite 200 PRO).

### 2.9 Wound Healing Assay

At 6 h after transfection of EGFP or p21 mRNA into T24 cells, a wound‐healing assay was performed. Briefly, a scratch was created in a confluent cell monolayer using a sterile pipette tip to generate a cell‐free gap. Cell migration into the gap was monitored and imaged at 0, 24, and 48 h. Images were captured using a Nikon ECLIPSE E800 microscope and analyzed with ImageJ software.

### 2.10 Colony Formation Assay

T24 cells at 60%–70% confluence in 6‐well plates were transfected with EGFP or p21 mRNA for 6 h, then harvested and re‐seeded into new 6‐well plates at a density of 500 cells/well. Cells were cultured in complete medium for 14 days, with medium replacement every 2–3 days. Colonies were subsequently washed with PBS, fixed with 4% paraformaldehyde for 15 min, and stained with 0.1% crystal violet for 20 min at room temperature. After gentle washing and air‐drying, colonies were photographed, and the number of colonies was quantified using ImageJ.

### 2.11 Flow Cytometry

Cells were seeded in 6‐well plates and transfected with mRNA at 60%–70% confluence. Twenty hours post‐transfection, cells were harvested, and apoptosis was assessed using the Annexin V‐FITC Apoptosis Detection Kit (LiankeBio, AP101) according to the manufacturer's protocol. Stained samples were analyzed on a CytoFLEX S flow cytometer (Beckman), and data were analyzed using FlowJo software.

### 2.12 Clinical Tissue Microarray and Immunohistochemistry

A bladder cancer tissue microarray (ZL‐BlaU961) containing 59 clinical cases was used to evaluate p21 protein expression in human bladder tissues. Formalin‐fixed, paraffin‐embedded sections were baked at 59°C for 60 min, deparaffinized in xylene, rehydrated through graded ethanol, and subjected to antigen retrieval in EDTA buffer (Weiao, WH1034) using heat‐induced epitope retrieval under high‐temperature and high‐pressure conditions. Endogenous peroxidase activity was blocked with 3% H_2_O_2_, followed by blocking with 5% BSA (Weiao, WH2051). Sections were incubated with an anti‐p21 primary antibody (Cell Signaling Technology, 2947, 1:50) at 4°C overnight, followed by HRP‐based detection using a universal immunohistochemistry kit (DAKO, K5007). Immunoreactivity was visualized with DAB (Weiao, WB0167) and counterstained with hematoxylin (Weiao, WH1145). Sections were then dehydrated, cleared, and mounted.

p21 staining was quantified by calculating H‐scores from five non‐overlapping fields per sample using the IHC Profiler plugin in ImageJ according to the formula: H‐score = 0 × negative + 1 × low positive + 2 × positive + 3 × high positive. Cases were grouped according to pathological stage (Tis, T1, T2, T3, and T4) for comparison. Statistical analyses were performed using GraphPad Prism 10.

### 2.13 Animal Studies

Tumor models were established using female BALB/c nude mice (5–6 weeks old). All animal experiments were conducted in compliance with the Animal Ethics and Welfare Guidelines of the Chinese Ministry of Public Health and were approved by the Animal Care and Experiment Committee of Shanghai Jiao Tong University School of Medicine (Accreditation No. JUMC2023‐047‐A).

### 2.14 Orthotopic Bladder Cancer Model

Mice were anesthetized with isoflurane, and a catheter was inserted into the bladder through the urethra. The bladder was drained, washed with PBS, and instilled with 50 μL of 0.5 M silver nitrate (AgNO₃) for 15 s to disrupt the urothelial barrier. After three PBS washes, 100 μL of T24‐Luc cell suspension (2 × 10^6^ cells) was instilled. A non‐invasive purse‐string suture was applied to the urethra for 2 h to improve tumor cell retention.

### 2.15 In Vivo Bioluminescence Imaging

Tumor burden was monitored using the IVIS Spectrum Imaging System (PerkinElmer). Tumor‐bearing mice were intraperitoneally injected with D‐luciferin (150 mg/kg). Mice were anesthetized via isoflurane inhalation, and imaging was performed 5 min post‐injection to capture the peak bioluminescent signal.

### 2.16 LNP Formulation and Characterization

Lipid nanoparticles (LNP) were formulated using a lipid mixture of SM‐102 (APExBIO, C1042), 1,2‐Distearoyl‐sn‐glycero‐3‐phosphocholine (DSPC) (Avanti Polar Lipids, 850 365), cholesterol (APExBIO, B1702), and PEG2000‐DMG (Avanti Polar Lipids, 880 151) at a molar ratio of 50:10:38.5:1.5, dissolved in ethanol. mRNA was diluted in 50 mM citrate buffer (pH 3.2). The lipid and mRNA solutions were mixed at a volumetric ratio of 1:3 using a Feather Microfluidic Chip (APExBIO, RM1002) on a NanoDispatcher L device (APExBIO, RM1001). The resulting formulations were diluted twofold with 50 mM citrate buffer (pH 3.2) and dialyzed against PBS (pH 7.4) for > 15 h using Slide‐A‐Lyzer dialysis cassettes (Thermo Fisher Scientific, 66 830). Formulations were concentrated using Amicon Ultra centrifugal filters (MilliporeSigma, UFC90008) and sterilized by filtration through a 0.22‐μm membrane. Particle size and polydispersity index (PDI) were measured using a Zetasizer (Malvern Panalytical). mRNA encapsulation efficiency and concentration were quantified using the Quant‐iT RiboGreen RNA Assay (Thermo Fisher Scientific, R11490).

### 2.17 In Vivo **LNP** Treatment and Imaging

For biodistribution studies, mice received an intravesical instillation of 10 μg Luc‐LNP and were subjected to in vivo bioluminescence imaging at 6, 24, 48, and 72 h after administration. At the indicated time points, mice were euthanized, and major organs, including the bladder, heart, lung, spleen, kidney, and liver, were collected for ex vivo bioluminescence imaging. Total flux in each organ was quantified using Living Image software.

For therapeutic efficacy studies, tumor‐bearing mice were randomized into three groups (*n* = 7) on day 3 post‐implantation. Mice received intravesical instillations of 100 μL PBS, EGFP‐LNP (10 μg in 100 μL PBS), or p21‐LNP (10 μg in 100 μL PBS) every 3 days for a total of 8 doses. Tumor growth was monitored by IVIS imaging after intraperitoneal D‐luciferin injection (150 mg/kg), and total flux (photons/s) in the bladder region was quantified.

### 2.18 Histology

Bladder tissues and major organs, including the heart, liver and kidney were harvested, fixed in 10% neutral buffered formalin, embedded in paraffin, and sectioned at 5 μm thickness. Sections were stained with Hematoxylin and Eosin (H&E) for pathological evaluation.

### 2.19 Statistical Analysis

Data are presented as mean ± standard deviation (SD) or standard error of the mean (SEM). Statistical analyses were performed using GraphPad Prism software. Comparisons among multiple groups were performed using one‐way ANOVA followed by Tukey's post hoc test. A *p*‐value < 0.05 was considered statistically significant.

## 3 Results

### 3.1 Rationale for Selecting *CDKN1A* /P21 as a Therapeutic Target in Bladder Cancer and Validation of P21 mRNA Expression

**FIGURE 1:**
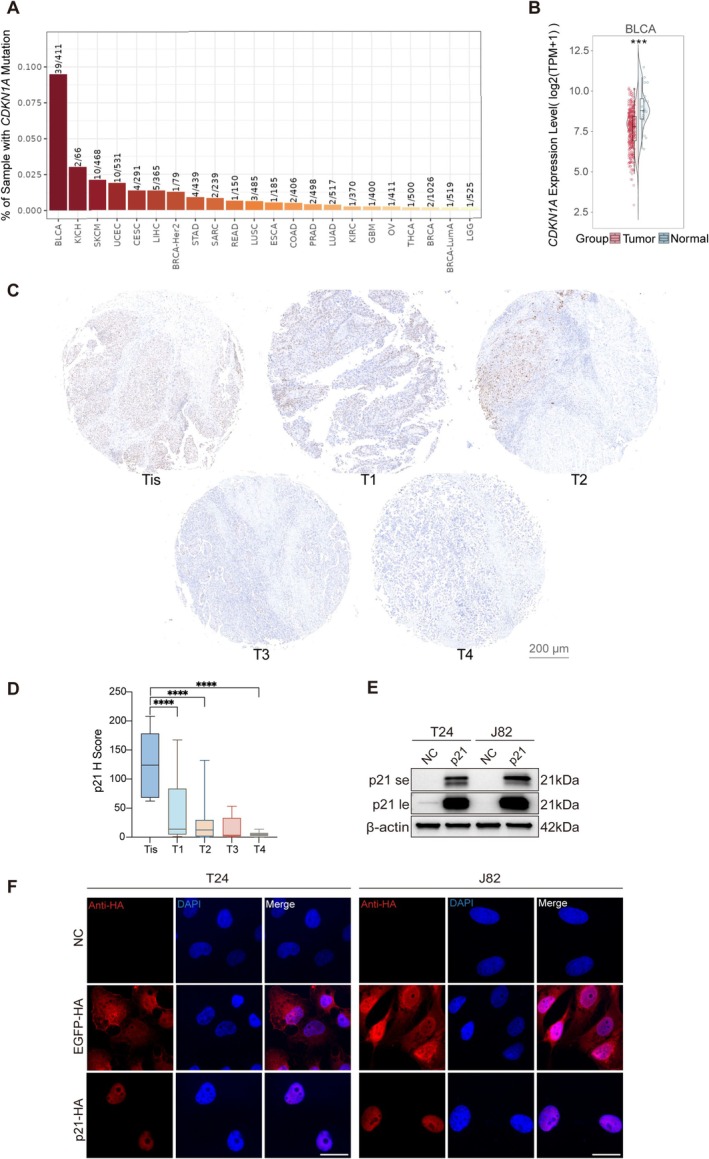
Rationale for selecting *CDKN1A*/p21 as a therapeutic target in bladder cancer and validation of p21 mRNA expression. (A) Pan‐cancer analysis of *CDKN1A* alteration frequencies based on TCGA datasets. The analysis was performed using the TIMER3 web server. (B) *CDKN1A* mRNA expression in bladder cancer and normal bladder tissues, shown as log2(TPM + 1), where TPM denotes transcripts per million. Data were analyzed using the TIMER3 web server. (C) Representative p21 immunohistochemical staining images from a bladder cancer tissue microarray stratified by pathological stage (Tis, T1, T2, T3, and T4). Scale bars, 200 μm. (D) Quantification of p21 H‐scores in Tis, T1, T2, T3, and T4 lesions. Compared with Tis lesions, T1, T2, T3, and T4 tumors showed significantly lower p21 H‐scores. Data are presented as mean ± SD. *p < 0.05, **p < 0.01 (one‐way ANOVA). (E) Western blot analysis of basal and p21 mRNA‐induced p21 protein expression in T24 and J82 bladder cancer cells. Basal p21 protein levels were very low in both cell lines, whereas transfection of p21 mRNA led to robust p21 expression. For p21 blots, both short‐exposure (se) and long‐exposure (le) images are shown. β‐Actin serves as a loading control. (F) Immunofluorescence analysis of EGFP‐HA and p21‐HA expression in T24 and J82 cells using an anti‐HA antibody. Nuclei were stained with DAPI. The p21‐HA signal was predominantly localized to the nucleus, whereas EGFP showed diffuse cytoplasmic distribution. Scale bars, 20 μm.

Given the recurrent inactivation of *CDKN1A*/p21 and the widespread disruption of the p53/cell‐cycle axis in bladder cancer, we evaluated the relevance of p21 loss using public datasets, clinical specimens, and bladder cancer cell lines. Pan‐cancer analysis based on TCGA datasets showed that bladder cancer exhibited one of the highest frequencies of *CDKN1A* alterations among the tumor types analyzed, affecting approximately 9%–10% of cases (Figure 1A). In addition, transcriptomic analysis showed that *CDKN1A* expression was significantly reduced in bladder cancer tissues compared with normal bladder tissues (Figure 1B), indicating recurrent downregulation of p21 during bladder tumorigenesis.

To further assess the clinical relevance of p21 loss at the protein level, we performed immunohistochemical staining of p21 in a bladder cancer tissue microarray and quantified p21 H‐scores according to pathological stage. Representative images showed a progressive reduction in p21 staining from Tis (carcinoma in situ) to more advanced lesions (Figure 1C). Quantitative analysis demonstrated that T1 (lamina propria invasion), T2 (muscularis propria invasion), T3 (perivesical soft tissue invasion), and T4 (invasion into adjacent organs or pelvic/abdominal wall) tumors all exhibited significantly lower p21 H‐scores than Tis lesions, whereas no significant differences were observed among the T1–T4 groups despite an overall decreasing trend with advancing stage (Figure 1D). These findings suggest that p21 downregulation is associated with bladder cancer progression.

To restore p21 function, we generated p21 mRNA using a T7 promoter‐based in vitro transcription (IVT) strategy, as illustrated in Figure S1A. We next examined basal p21 expression in bladder cancer cell lines and found that both T24 and J82 cells expressed very low basal p21 protein levels. In contrast, transfection of p21 mRNA led to robust p21 protein expression in both cell lines, as demonstrated by western blotting (Figure 1E). Immunofluorescence analysis further showed that the translated p21 protein was predominantly localized in the nucleus, whereas the EGFP control displayed diffuse cytoplasmic distribution (Figure 1F). Together, these genomic, histopathological, and cellular data support the selection of *CDKN1A*/p21 as a tumor suppressor replacement target in bladder cancer and confirm that IVT‐derived p21 mRNA can be efficiently translated to produce properly localized p21 protein in bladder cancer cells.

### 3.2 P21 mRNA Suppresses Proliferation in Bladder Cancer Cells

**FIGURE 2:**
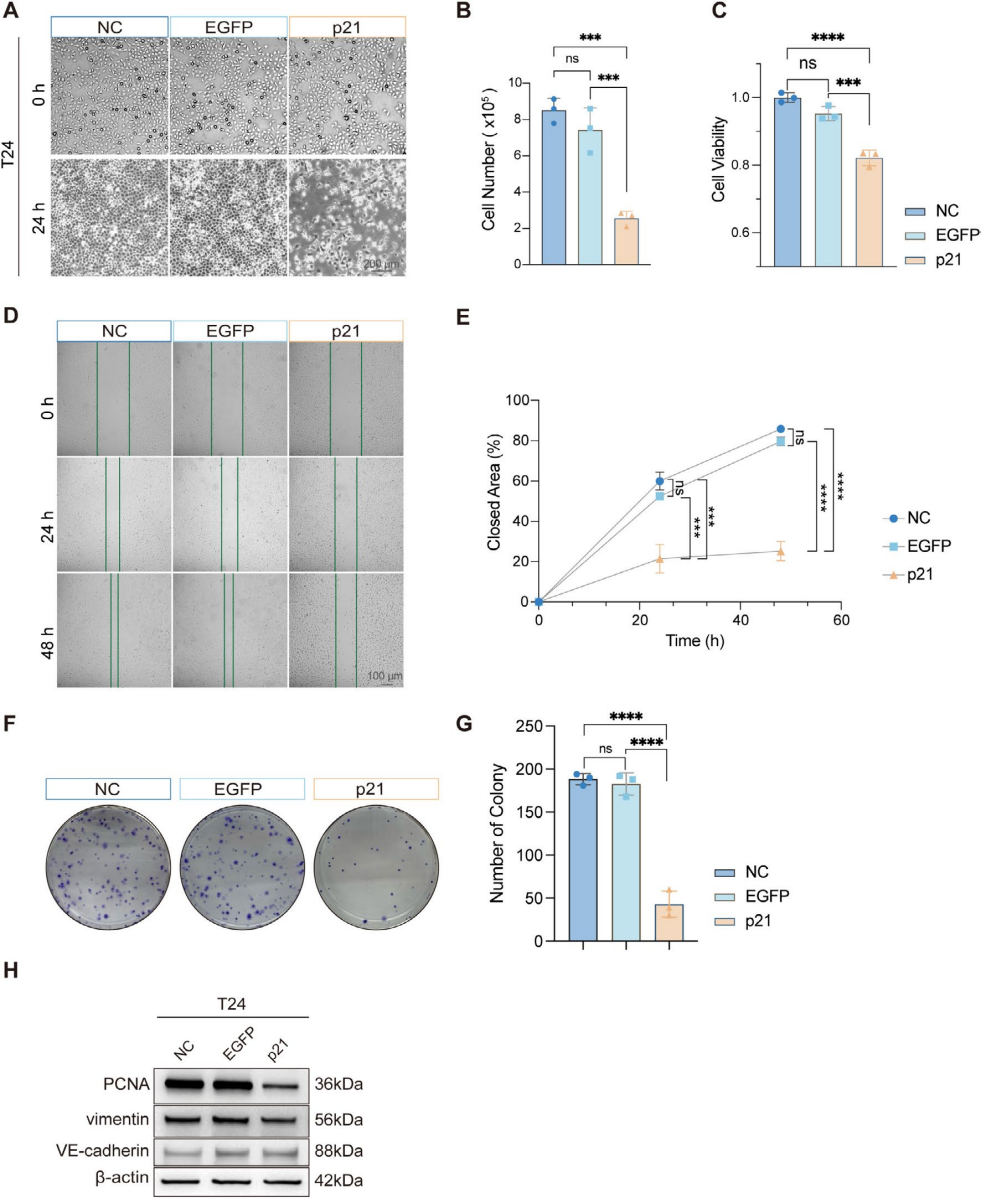
P21 mRNA suppresses proliferation in bladder cancer cells. (A) Representative images of T24 cells in the NC, EGFP mRNA, and p21 mRNA groups at 0 h and 24 h post‐transfection. Scale bars, 200 μm. (B) Quantification of cell numbers 24 h after mRNA transfection. (C) Cell viability was assessed by CCK‐8 assay at 24 h post‐transfection. (D) Representative images from wound‐healing assays performed at 0 h, 24 h, and 48 h after scratch induction in T24 cells of the indicated groups. Scale bars, 100 μm. (E) Quantification of wound closure over time. (F) Representative images of colony formation assays in the indicated groups. (G) Quantification of colony numbers from (F). (H) Western blot analysis of proliferation‐ and invasion‐related markers, including PCNA, Vimentin, and VE‐cadherin, in T24 cells following mRNA transfection. β‐Actin serves as a loading control.

To assess the functional impact of p21 restoration, T24 bladder cancer cells were transfected with p21 mRNA or EGFP mRNA. While all groups exhibited comparable cell density at baseline, p21 mRNA‐transfected cells displayed pronounced morphological changes at 24 h, including cell rounding, detachment, and reduced density, whereas NC and EGFP cells reached near confluence (Figure 2A). Quantitative cell counting showed inhibition of T24 cell proliferation following p21 mRNA treatment (Figure 2B).

To further assess cell viability, cells were re‐counted 6 h post‐transfection—when mRNA expression had reached peak levels—and reseeded for Cell Counting Kit‐8 (CCK‐8) assay. At 24 h, EGFP mRNA had no significant effect on viability, whereas p21 mRNA markedly reduced cell viability relative to controls (Figure 2C).

Cell migratory capacity was assessed using a wound‐healing assay. Scratches were introduced 6 h after transfection, and wound closure was monitored at 0, 24, and 48 h. Compared with the NC and EGFP groups, p21 mRNA‐transfected cells displayed markedly delayed wound closure over time (Figure 2D,E). By 48 h, NC and EGFP cells achieved > 80% wound recovery, whereas p21 mRNA‐treated cells exhibited only ~20% closure. Because wound‐healing measurements can be influenced by both cell migration and cell proliferation, and because p21 restoration exerted a strong anti‐proliferative effect in parallel assays, the delayed wound closure observed here is interpreted primarily as impaired closure capacity rather than as definitive evidence of a direct anti‐migratory effect. Consistent with this growth‐suppressive phenotype, colony formation assays showed a substantial reduction in clonogenic capacity following p21 mRNA transfection (Figure 2F,G). Together, these data indicate that p21 mRNA markedly suppresses the proliferative potential of T24 bladder cancer cells and delays wound closure in vitro.

To further characterize the effect of p21 restoration at the molecular level, we assessed biomarkers associated with proliferation and invasion. Western blot analysis showed that proliferating cell nuclear antigen (PCNA), a canonical proliferation marker, was markedly downregulated after p21 mRNA transfection (Figure 2H), in agreement with the observed inhibition of cell growth and clonogenicity. By contrast, the migration/invasion‐related markers Vimentin and VE‐cadherin did not show obvious changes (Figure 2H). These findings suggest that p21 restoration primarily exerts an anti‐proliferative effect, while its impact on migration or invasion‐associated programs appears limited under the conditions tested.

### 3.3 P21 mRNA Induces Cell‐Cycle Arrest, DNA Damage, and Apoptosis in Vitro

**FIGURE 3:**
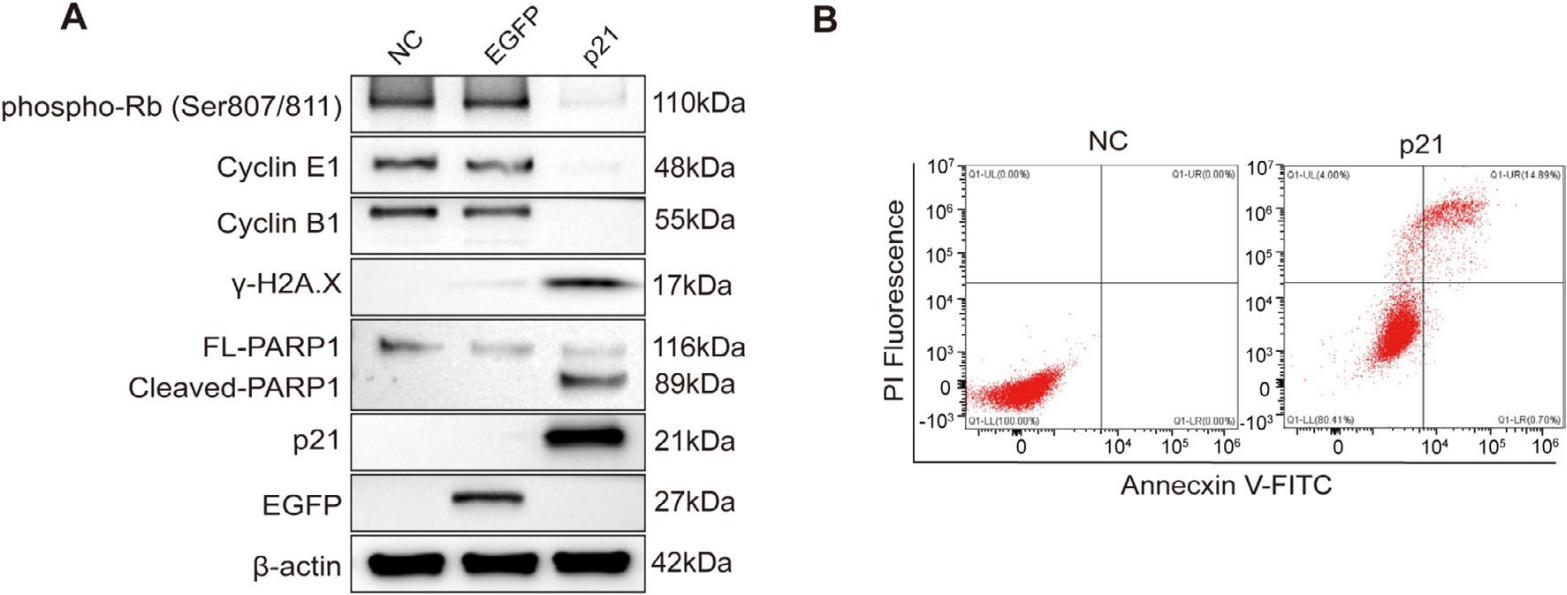
P21 mRNA induces cell‐cycle arrest, DNA damage, and apoptosis in vitro. (A) Immunoblot analysis of cell cycle‐, DNA damage‐, and apoptosis‐related proteins in T24 cells following mRNA transfection, including phospho‐Rb (Ser807/811), Cyclin E, Cyclin B, γ‐H2A.X, and PARP1. β‐Actin serves as a loading control. (B) Flow cytometric analysis of apoptosis using Annexin V–fluorescein isothiocyanate (FITC)/PI staining in T24 cells after mRNA transfection.

To elucidate the molecular mechanisms underlying p21‐mediated phenotypes, we performed immunoblot analysis. Consistent with p21's role as a potent CDK inhibitor, p21 overexpression led to a significant reduction in phosphorylated retinoblastoma protein (Rb) (Ser807/811) (Figure 3A). This hypo‐phosphorylation of Rb prevents the release of E2F transcription factors, thereby repressing E2F target genes, including *Cyclin E* [19]. Accordingly, we observed a sharp decline in Cyclin E protein level (Figure 3A). Cyclin B levels were also significantly downregulated, suggesting that the inhibitory effect of p21 extends beyond the G1/S transition and also influences G2/M‐associated cell‐cycle control.

Notably, p21 mRNA‐transfected cells exhibited elevated γ‐H2A.X levels, indicating increased DNA double‐strand break‐associated signaling, consistent with replication stress induced by abrupt cell‐cycle arrest (Figure 3A). In agreement with these observations, apoptosis was evidenced by increased PARP1 cleavage (Figure 3A) and a higher proportion of Annexin V/PI‐positive cells detected by flow cytometry (Figure 3B). Collectively, these results indicate that p21 mRNA‐mediated restoration of p21 effectively enforces cell‐cycle arrest and triggers apoptotic cell death in bladder cancer cells.

### 3.4 Formulation and Characterization of mRNA‐Loaded Lipid Nanoparticles

**FIGURE 4:**
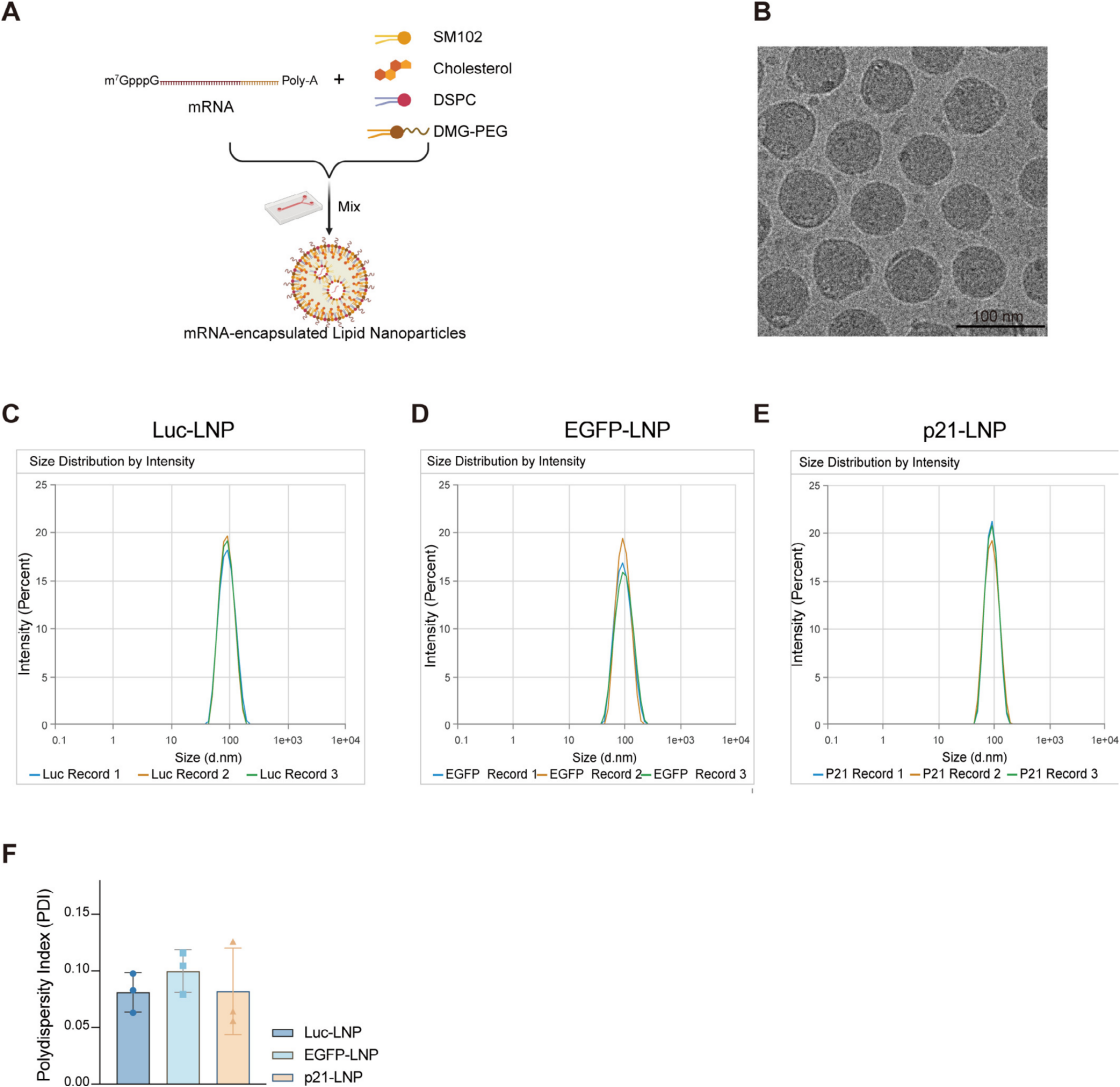
Formulation and characterization of mRNA‐loaded lipid nanoparticles. (A) Schematic illustration of mRNA encapsulation into lipid nanoparticles (LNP) using a microfluidic mixing platform. mRNA was formulated with SM‐102, cholesterol, DSPC, and DMG‐PEG to generate mRNA‐loaded LNP. (B) Transmission electron microscopy image showing the uniform spherical morphology of p21‐LNP. Scale bar, 100 nm. (C–E) Dynamic light scattering analysis of particle size distribution for Luc‐LNP, EGFP‐LNP, and p21‐LNP, respectively, demonstrating narrow and reproducible size distributions. (F) Polydispersity index (PDI) of Luc‐, EGFP‐, and p21‐LNP, indicating high batch uniformity. Data are presented as mean ± SD.

To achieve efficient in vivo mRNA delivery, we encapsulated mRNA into lipid nanoparticles (LNP) using a microfluidic mixing platform to ensure rapid and homogeneous self‐assembly (Figure 4A). Structural characterization by transmission electron microscopy (TEM) showed that the resulting p21‐LNP exhibited a uniform and well‐dispersed spherical morphology, with particle diameters slightly below 100 nm (Figure 4B).

Consistent with the TEM observations, dynamic light scattering (DLS) analysis further confirmed that luciferase‐ (Luc‐), EGFP‐, and p21‐LNP all displayed narrow size distributions with high batch uniformity (Figure 4C–E). In addition, the polydispersity index (PDI) remained below 0.15 across all formulations (Figure 4F), indicating a highly homogeneous nanoparticle population. Together, these results demonstrate that the microfluidic formulation strategy yields stable mRNA‐LNP preparations with favorable physicochemical properties for subsequent in vivo administration.

### 3.5 Intravesical Administration of Luc‐LNP Enables Localized Expression With Limited Systemic Distribution and No Obvious Acute Organ Toxicity

**FIGURE 5:**
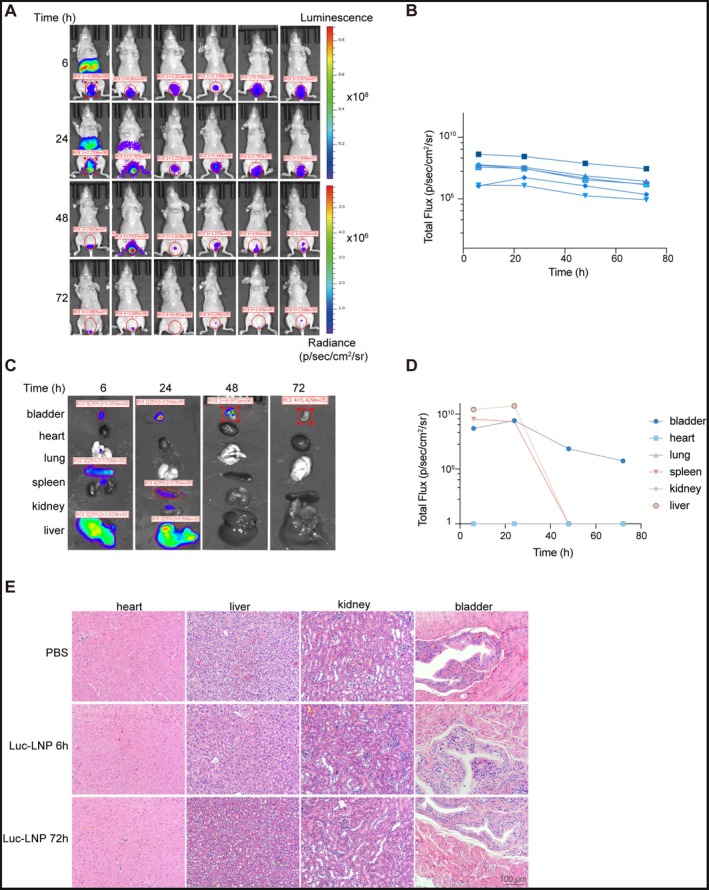
Intravesical administration of Luc‐LNP enables localized expression with limited systemic distribution and no obvious acute organ toxicity. (A) Representative in vivo bioluminescence imaging of mice at 6 h, 24 h, 48 h, and 72 h after intravesical administration of Luc‐LNP. (B) Quantification of total bioluminescent flux in the bladder region over time. Each colored line represents an individual mouse. (C) Representative ex vivo bioluminescence imaging of six organs, including the bladder, heart, lung, spleen, kidney, and liver, collected at 6 h, 24 h, 48 h, and 72 h after intravesical administration of Luc‐LNP. (D) Quantification of luciferase signal intensity in the bladder, heart, lung, spleen, kidney, and liver over time. Bladder signals remained detectable up to 72 h, whereas transient signals in the spleen and liver were mainly observed at 6 h and 24 h and became undetectable after 48 h. (E) Representative H&E‐stained sections of the heart, liver, kidney, and bladder collected from mice treated with PBS, or examined at 6 h and 72 h after intravesical administration of Luc‐LNP, showing no obvious histopathological abnormalities. Scale bars, 100 μm.

To assess whether intravesical instillation could serve as an effective route for local delivery of mRNA‐LNP, we first used Luc‐LNP as a reporter system. Following intravesical administration of 10 μg Luc‐LNP into the mouse bladders, bioluminescence was detected in the bladder region (Figure 5A). Time‐course analysis showed that the luciferase signal peaked at approximately 6 h after instillation and remained detectable for up to 72 h, demonstrating sustained local expression after bladder administration (Figure 5A,B).

To further evaluate the in vivo distribution of the formulation, mice were sacrificed at 6, 24, 48, and 72 h after intravesical administration of Luc‐LNP, and six organs, including the bladder, heart, lung, spleen, kidney, and liver, were collected for ex vivo bioluminescence analysis. The strongest and most sustained signal was detected in the bladder, where luciferase activity remained measurable up to 72 h (Figure 5C,D). At 6 h and 24 h, relatively stronger transient signals were also observed in the spleen and liver, whereas these signals became undetectable after 48 h. In contrast, the heart, lung, and kidney showed minimal or negligible signal throughout the observation period (Figure 5C,D). These results indicate that intravesical delivery of Luc‐LNP enables prolonged local expression in the bladder with limited and transient distribution to distant organs.

To further assess acute systemic safety, H&E staining was performed on major organs after intravesical administration of PBS or Luc‐LNP. No obvious histopathological abnormalities, degenerative changes, or inflammatory lesions were observed in the heart, liver, kidney, or bladder at either 6 h or 72 h after treatment (Figure 5E), supporting the favorable short‐term tolerability of intravesical LNP delivery.

### 3.6 Intravesical P21‐LNP Treatment Suppresses Orthotopic Bladder Tumor Growth

**FIGURE 6:**
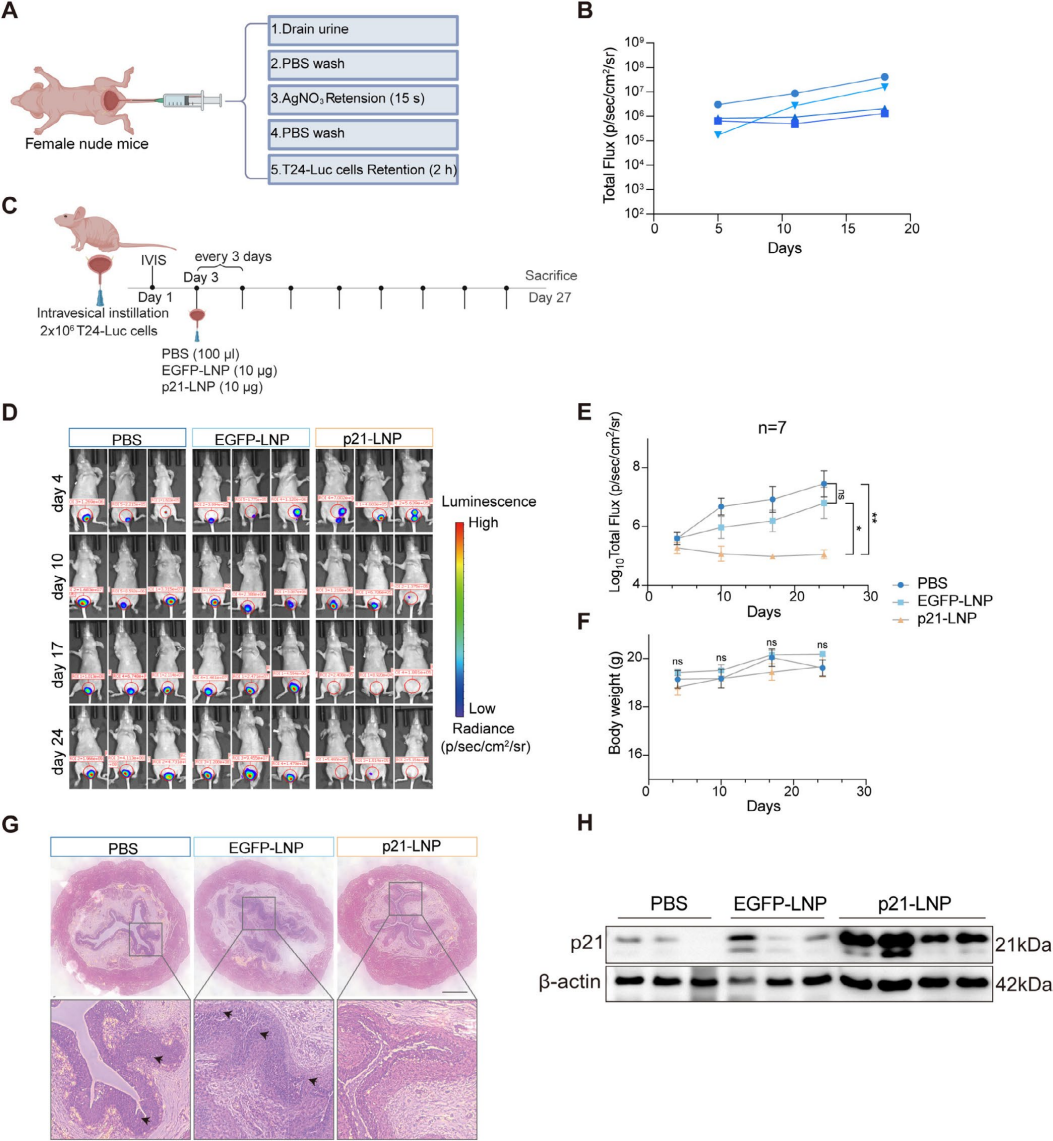
Intravesical p21‐LNP treatment suppresses orthotopic bladder tumor growth in vivo. (A) Schematic illustration of the orthotopic T24‐Luc bladder cancer model. (B) Quantification of total bioluminescent flux during orthotopic tumor establishment, showing progressive tumor growth in the bladder. Each colored line represents an individual mouse. Representative in vivo images of bladder‐localized tumors are shown in Figure S2A. (C) Schematic overview of the in vivo treatment regimen. Tumor‐bearing mice received intravesical instillation of PBS, EGFP‐LNP, or p21‐LNP every 3 days starting on day 3 post‐implantation. (D) Representative bioluminescence images of mice from each treatment group at the indicated time points. (E) Quantification of tumor burden based on total bioluminescent flux over time (*n* = 7 per group). (F) Body weight monitoring during treatment, showing no significant differences among groups. (G) Representative H&E‐stained bladder sections from each treatment group. Scale bars, 100 μm. (H) Western blot analysis of p21 protein levels in bladder tissues from each treatment group. β‐Actin serves as a loading control.

To evaluate therapeutic efficacy in vivo, we established an orthotopic bladder cancer model using luciferase‐labeled T24‐Luc cells (Figure 6A). Bladder mucosa was transiently disrupted with 0.5 M silver nitrate to facilitate tumor cell adherence, followed by intravesical instillation of T24‐Luc cells. Bioluminescence imaging on days 5, 11, and 18 revealed progressively increasing signals confined to the bladder, indicating stable tumor engraftment and continuous growth (Figures 6B and S2A).

We next used this orthotopic model to assess the therapeutic efficacy of intravesical p21‐LNP treatment. Tumor‐bearing mice were randomized to receive intravesical instillation of 100 μL per mouse of PBS, EGFP‐LNP (10 μg), or p21‐LNP (10 μg) every 3 days for a total of 8 doses, beginning on day 3 after tumor implantation (Figure 6C). Tumor progression was monitored by bioluminescence imaging. While tumor burden remained comparable across all groups on day 4, a distinct divergence in growth kinetics emerged by day 10 (Figure 6D,E). Compared with the PBS and EGFP‐LNP groups, the p21‐LNP‐treated group exhibited significantly reduced bioluminescent tumor signals, and this suppressive effect was maintained until the study endpoint (Figure 6D,E).

Importantly, no significant body weight loss was observed during the treatment period (Figure 6F), suggesting good tolerability of repeated intravesical administration. Histological analysis further supported the imaging results. Bladders from the PBS and EGFP‐LNP groups showed marked urothelial thickening, invasive tumor growth, and nuclear atypia, whereas bladders from the p21‐LNP‐treated group largely retained normal urothelial architecture and showed no obvious tumor‐associated thickening or invasion (Figure 6G). At 48 h after the final intravesical administration on day 24, bladder tissues were collected for western blot analysis, which further confirmed that p21 protein was significantly elevated in the p21‐LNP‐treated group (Figure 6H), demonstrating successful local restoration of p21 expression in vivo.

Together, these findings show that intravesical delivery of p21 mRNA‐LNP effectively restores p21 expression and suppresses orthotopic bladder tumor progression.

## 4 Discussion

In this study, we demonstrate that intravesical delivery of p21 mRNA‐loaded lipid nanoparticles is a feasible and effective tumor suppressor replacement strategy for bladder cancer. By integrating target selection, mRNA engineering, nanoparticle formulation, in vitro functional analysis, biodistribution assessment, and orthotopic in vivo validation, we establish a localized mRNA therapeutic framework for restoring tumor suppressor activity in the bladder.

A central strength of this work is the rationale for selecting *CDKN1A/*p21 as the therapeutic target. Public datasets and prior genomic studies have identified recurrent *CDKN1A* alterations and downregulation in bladder cancer, implicating p21 loss as part of a broader p53/cell‐cycle dysregulation program [13, 14]. Consistent with this, our tissue microarray analysis showed that p21 protein expression was significantly lower in T1–T4 lesions than in Tis, suggesting that p21 downregulation is associated with disease progression. Together with the very low basal p21 expression observed in T24 and J82 cells, these data support p21 loss as a biologically relevant vulnerability in bladder cancer.

Functionally, IVT‐derived p21 mRNA restored robust p21 expression from a very low basal background and generated the expected nuclear localization in bladder cancer cells. This was followed by marked reductions in cell number, viability, wound closure, and clonogenic growth. At the molecular level, p21 restoration reduced Rb phosphorylation, downregulated Cyclin E, Cyclin B, and PCNA, increased γ‐H2A.X, and promoted PARP1 cleavage and Annexin V/PI positivity. These findings are consistent with a model in which restored p21 suppresses bladder cancer cell growth primarily through potent cell‐cycle inhibition, followed by DNA damage‐associated stress and apoptosis [15, 16]. The lack of obvious changes in Vimentin and VE‐cadherin further suggests that p21 restoration primarily exerts an anti‐proliferative effect, while its impact on migration‐ or invasion‐associated programs appears limited under the conditions tested.

The therapeutic feasibility of this strategy depends on efficient local mRNA delivery. Here, we formulated p21 mRNA into LNP with uniform spherical morphology, narrow size distribution, and low PDI, consistent with high‐quality nanoparticles suitable for intravesical administration [8, 10, 20]. Using luciferase mRNA as a reporter, we further showed that intravesical administration resulted in strong bladder‐localized protein expression lasting up to 72 h. Ex vivo organ imaging demonstrated that the strongest and most persistent signal remained confined to the bladder, whereas only transient signals were detected in the spleen and liver at early time points. These findings indicate that intravesical LNP delivery can achieve effective local expression with limited and short‐lived systemic distribution.

The bladder provides a uniquely favorable setting for localized RNA therapy [2, 11, 12]. Its hollow anatomy and established catheter‐based instillation route allow direct exposure of therapeutic agents to urothelial tumors while minimizing systemic dissemination [11, 12]. This feature is particularly compatible with the transient expression profile of mRNA, which may be disadvantageous in systemic settings but becomes advantageous when repeated local dosing is clinically feasible. In this context, p21 is especially attractive because even short‐term restoration can rapidly engage downstream checkpoint machinery and produce sustained antiproliferative effects [15, 16].

In the orthotopic T24‐Luc bladder cancer model, repeated intravesical administration of p21‐LNP significantly suppressed tumor growth, preserved bladder histological architecture, and restored p21 protein expression in vivo. These findings are notable because the T24 model harbors defects in the p53 pathway, suggesting that direct p21 replacement can bypass upstream impairment and restore a critical downstream tumor‐suppressive function.

Safety is an important consideration for translation. In the present study, intravesical administration of Luc‐LNP resulted in predominantly bladder‐confined reporter expression, with no persistent off‐target signal after 48 h. Histological examination of the heart, liver, kidney, and bladder showed no obvious pathological abnormalities after Luc‐LNP administration, and repeated intravesical treatment with p21‐LNP did not cause significant body weight loss. Together, these findings support the favorable short‐term tolerability of intravesical LNP delivery [8, 21].

Several limitations should be noted. First, although the orthotopic T24 model recapitulates important features of bladder tumor growth and intravesical treatment, it does not fully reflect the molecular heterogeneity of patient tumors. Second, because the present in vivo *s*tudies were performed in immunodeficient mice, the impact of p21 restoration on the tumor immune microenvironment could not be evaluated. Increasing evidence suggests that cell‐cycle‐targeted perturbation and apoptosis may be accompanied by the release of damage‐associated molecular patterns and tumor‐associated antigens, potentially reshaping antitumor immunity [22, 23]. Third, while the biodistribution data and short‐term histological analysis support limited systemic exposure and favorable tolerability, they do not constitute a full pharmacokinetic or long‐term toxicology assessment. Further optimization of LNP composition may also improve urothelial penetration, tumor selectivity, and therapeutic durability [12].

In conclusion, this study establishes intravesical delivery of p21 mRNA‐loaded lipid nanoparticles as a promising localized therapeutic strategy for bladder cancer. By combining a clinically grounded target selection rationale, efficient tumor suppressor restoration, local nanoparticle‐mediated mRNA delivery, and in vivo therapeutic validation, our work provides strong support for localized mRNA‐based tumor suppressor replacement therapy as a clinically relevant approach for bladder cancer treatment.

## Author Contributions

Y.X. and C.W. conceived and designed the study. J.Z. performed the in vitro experiments. C.W. supervised the establishment of the animal tumor models. J.Z. and Z.C. jointly completed the in vivo experiments. Data analysis and manuscript writing were carried out by J.Z. and Y.X. C.W. contributed to manuscript revision.

## Funding

This work was supported by the Science and Technology Innovation Plan of Shanghai Science and (STCSM) Technology Commission, 23S11900300.

## Conflicts of Interest

The authors declare no conflicts of interest.

## Supporting information


**Figure S1:** In vitro transcription workflow for generating p21 mRNA.
(A) Schematic overview of the T7 promoter‐based in vitro transcription (IVT) workflow used to generate p21 mRNA in this study.

**Figure S2:** Validation of the orthotopic T24‐Luc bladder cancer model.
(A) Representative in vivo bioluminescence images showing localization of orthotopic T24‐Luc bladder tumors in mice.

## Data Availability

If derived from public domain information.
