# Supplementary figures and images for: Intravesical Delivery of P21 mRNA–Loaded Lipid Nanoparticles as a Tumor Suppressor Replacement Therapy for Bladder Cancer
Source: FASEB J. 2026 May 17;40:e71904. doi: 10.1096/fj.202600049R (PMC13181305; doi:10.1096/fj.202600049R)

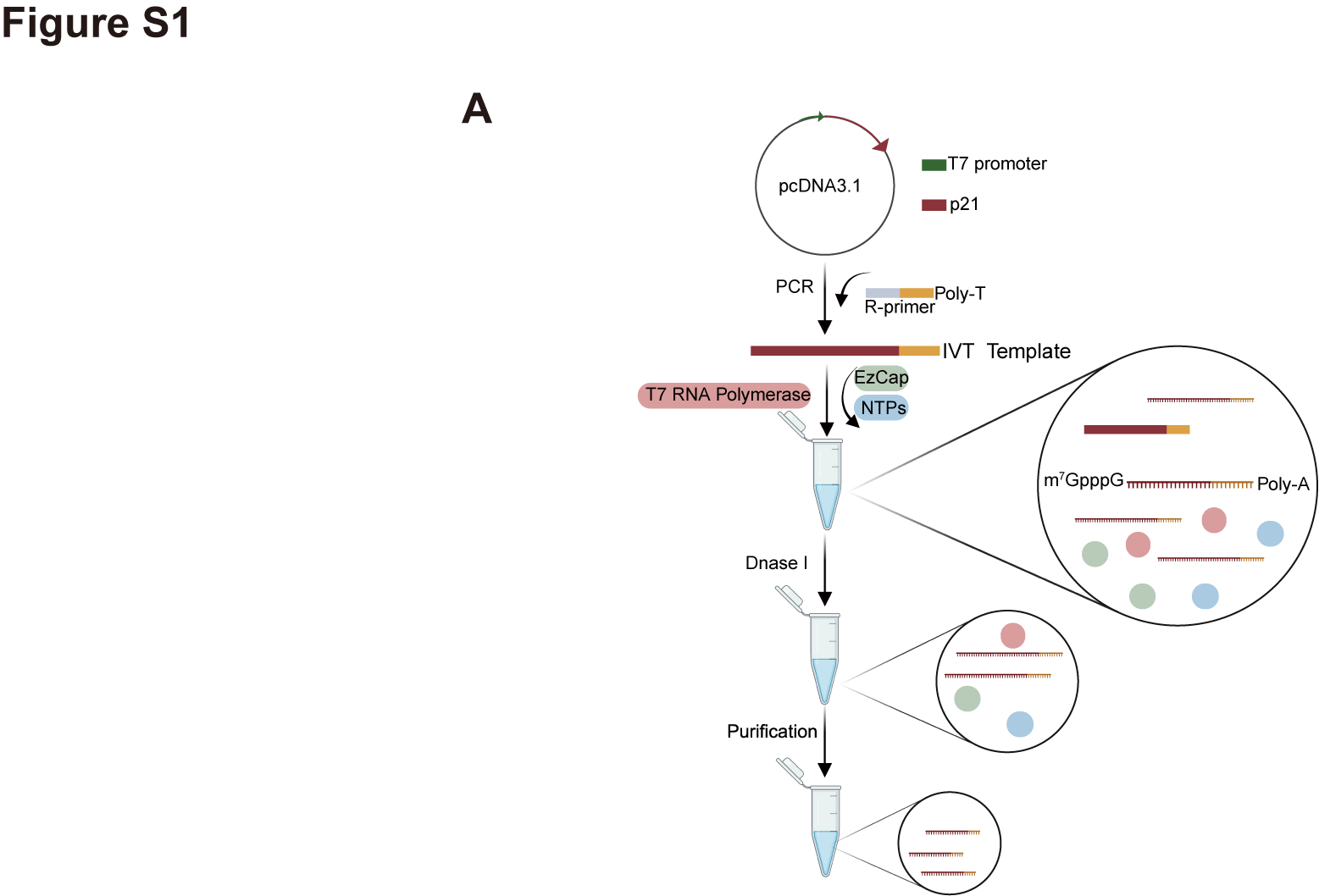

Supplement: Supplementary file 1 — Figure S1: In vitro transcription workflow for generating p21 mRNA. (A) Schematic overview of the T7 promoter‐based in vitro transcription (IVT) workflow used to generate p21 mRNA in this study. [file FSB2-40-e71904-s002.tif]

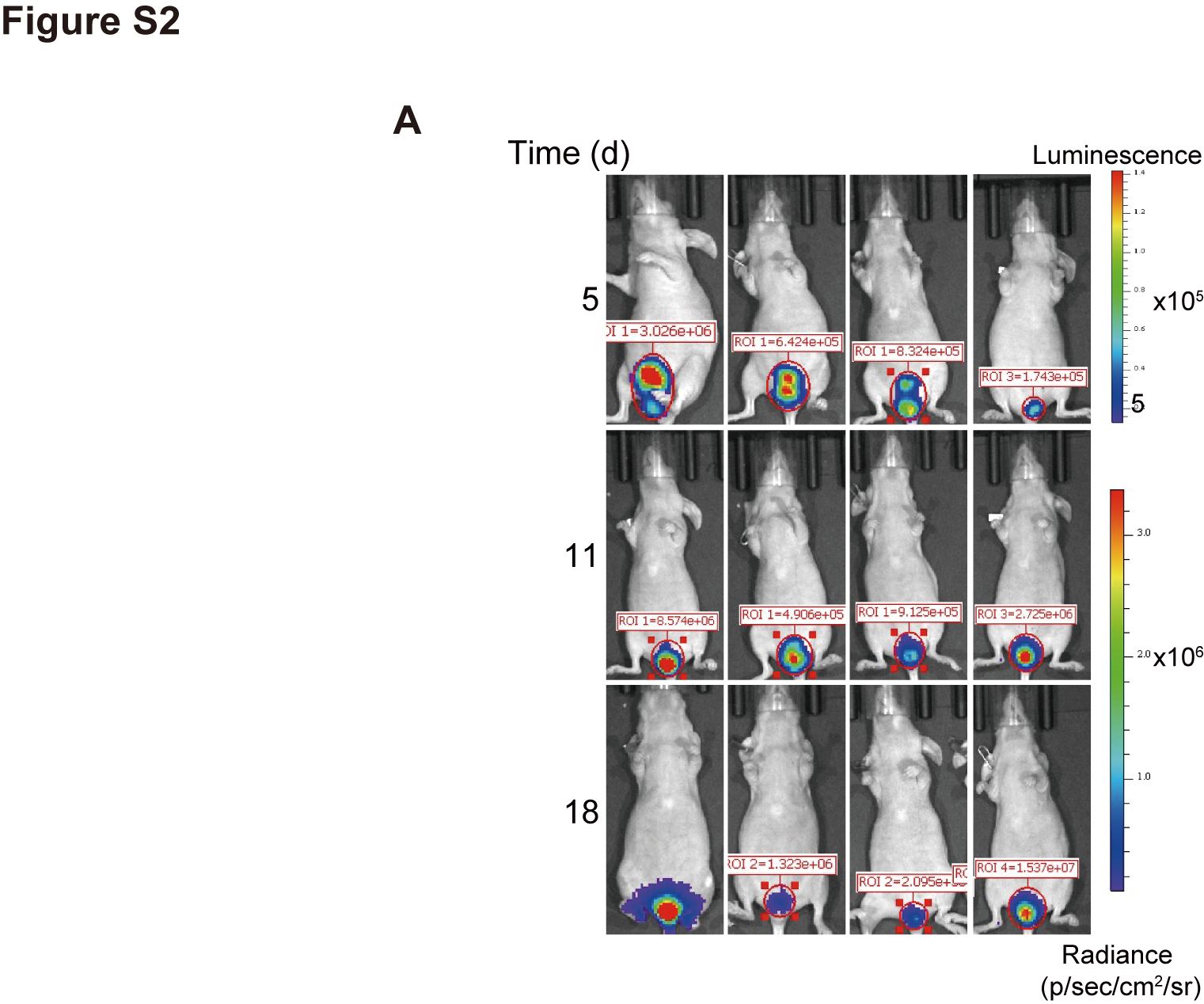

Supplement: Supplementary file 2 — Figure S2: Validation of the orthotopic T24‐Luc bladder cancer model. (A) Representative in vivo bioluminescence images showing localization of orthotopic T24‐Luc bladder tumors in mice. [file FSB2-40-e71904-s001.tif]
